# Cushing syndrome as presenting symptom of calcifying nested stromal–epithelial tumor of the liver in an adolescent boy: a case report

**DOI:** 10.1186/s13256-016-0951-2

**Published:** 2016-06-16

**Authors:** V. B. Weeda, Ph. R. de Reuver, H. Bras, J. Zsíros, W. H. Lamers, D. C. Aronson

**Affiliations:** 1 Tytgat Institute at the Academic Medical Center, Meibergdreef 69-71, S-building, 1105 BK Amsterdam, The Netherlands; 2 Pediatric Surgical Center of Amsterdam, Meibergdreef 9, 1105 AZ PO BOX 22660, Amsterdam, The Netherlands; 3 Department of Pathology, Academic Medical Center, Meibergdreef 9, 1105 AZ Amsterdam, The Netherlands; 4 Division of Pediatric Oncology, Emma Children’s Hospital at the Academic Medical Center, Meibergdreef 9, 1105 AZ Amsterdam, The Netherlands; 5 Department of Paediatric Surgery, Leeds Children’s Hospital at the Leeds Teaching Hospitals NHS Trust, Leeds, West Yorkshire LS1 3EX UK

**Keywords:** Ectopic ACTH production, Calcifying nested stromal–epithelial tumor of the liver, Desmoplastic nested spindle cell tumor of liver, Liver tumor, Pediatric

## Abstract

**Background:**

Ectopic adrenocorticotropic hormone-producing primary liver tumors are rare, especially in children. We report the case of an adolescent boy of mixed Dutch and Moroccan descent with an adrenocorticotropic hormone-producing calcifying nested stromal–epithelial tumor with long-term follow-up. Thus far, only two such cases have been reported.

**Case presentation:**

A 16-year-old boy of mixed Dutch and Moroccan descent presented with Cushing syndrome and a palpable abdominal mass. A calcifying nested stromal–epithelial tumor was diagnosed. Postoperatively, his plasma adrenocorticotropic hormone concentration normalized. He remains in complete remission 13 years after tumor resection.

**Conclusions:**

Calcifying nested stromal–epithelial tumor should be in the differential diagnosis of liver tumors, especially if associated with Cushing syndrome as significant morbidity and mortality may be associated. Literature on the topics involved is comprehensively reviewed.

## Background

Calcifying nested stromal–epithelial tumor (CNSET) of the liver is a rare tumor consisting of nests of spindled epithelioid cells with a potential for calcification [1]. Since its description, 37 cases have been reported (reviewed in [1–8]). Three small series of four, six and nine cases respectively, were reported as “Desmoplastic nested spindle cell tumor of the liver” [3] or as (calcifying) nested stromal cell tumor: (C)NSET [1, 5]. A similar tumor was reported as “Ossifying malignant mixed epithelial and stromal tumor of the liver” [2]. Patients’ age at diagnosis ranged from 1 to 34 years. Nine cases of CNSET with ectopic adrenocorticotropic hormone (ACTH) production have been described (reviewed in [8]), with six cases in adolescent females. Ectopic ACTH production is reported sporadically in liver tumors, for instance in hepatoblastoma [9], and combined hepatocellular carcinoma (HCC) and carcinoid [10]. In this case report a 16-year-old boy with Cushing syndrome due to CNSET is presented; it is the third report of such a case in an adolescent male with the longest follow-up (13 years). After tumor resection, his Cushing syndrome disappeared.

## Case presentation

**Fig. 1:**
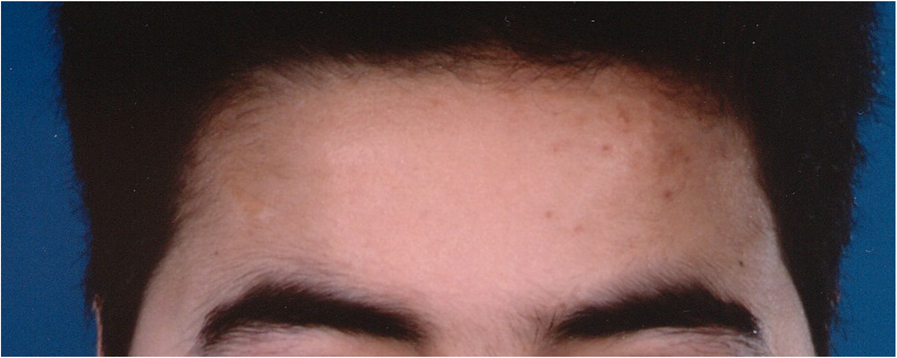
Detail of the patient’s anonymized face showing moon face, hyperpigmentation, and hirsutism

**Fig. 2:**
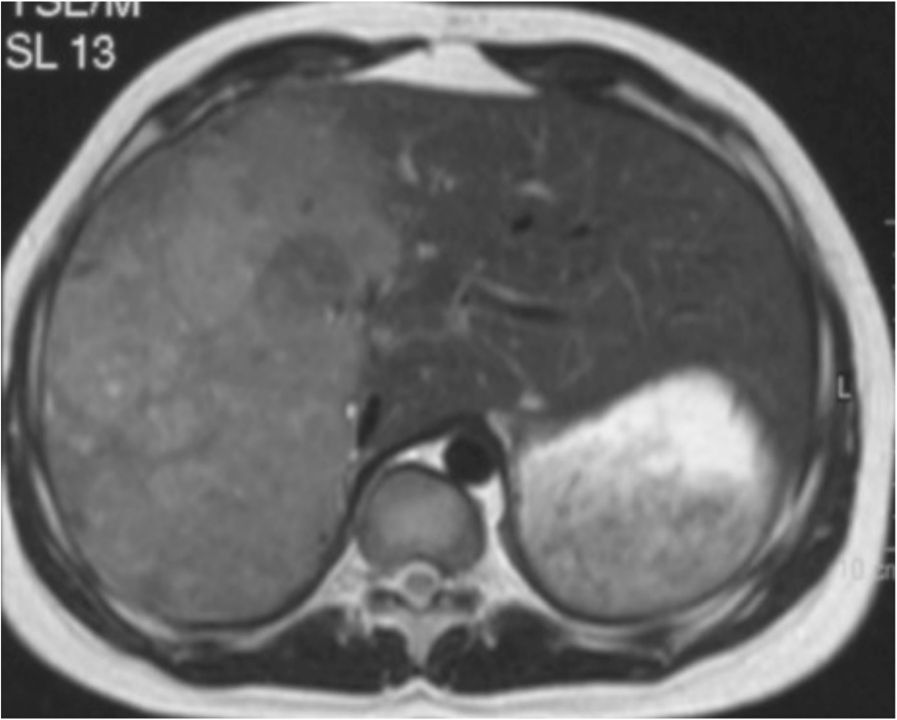
Image of magnetic resonance imaging scan showing a large mass in segments 4 to 8 of the liver

A 16-year-old boy of mixed Dutch and Moroccan descent was admitted to our pediatric department with a 2-month history of weight gain, distended abdomen, acne, and hypertension. On examination, he had a moon face, hirsutism, and hyperpigmentation (Fig. 1). His blood pressure was 142/88 mmHg. An unpainful mass was palpated in his upper right abdominal quadrant. Magnetic resonance imaging (MRI) showed a large mass in his liver segments 4 to 8 (Fig. 2), without evidence of distant metastases. Laboratory findings showed a white blood cell count of 17.9×10^9^/L and 431×10^9^/L platelets. Electrolytes, creatinine, and glucose levels were normal. His plasma gamma-glutamyltransferase concentration was mildly elevated (145 U/L). Endocrine findings were characteristic for Cushing syndrome, with plasma ACTH and cortisol concentrations of 285 ng/L and 785 nmol/L, respectively. His hypertension was treated with a calcium antagonist and ketoconazole therapy was administered to suppress steroid hormone synthesis. MRI of his brain ruled out an ACTH-producing pituitary tumor. A liver biopsy was performed (described in the Pathology subsection). Since the tumor appeared resectable, trisegmentectomy was performed. His ACTH levels normalized within 2 days after surgery. Postoperative complications consisted of bile leakage and ischemic biliary stenosis with jaundice. Introduction of a stent via endoscopic retrograde cholangiopancreatography was unsuccessful and percutaneous transhepatic cholangiography drainage was established. A month after surgery, a Roux-en-Y diversion with biliojejunostomy was performed to resolve the obstruction. The most recent follow-up imaging (an abdominal ultrasound performed in January 2013) showed no signs of recurrence. Thirteen years after resection he is doing well and remains in complete remission.

### Pathology

Core needle biopsy showed irregular fields of epithelial cell nests in a desmoplastic stroma, and dissociation of cells, yielding to pseudovascular spaces. Cells varied from epithelioid to spindle shaped. Immunohistochemistry was positive for cytokeratin 19, anticytokeratin, neural cell adhesion molecule, smooth muscle actin (SMA) and ACTH. No mitoses and no distinctive cellular atypia were found. Stromal cells were SMA-positive. A small cell desmoplastic tumor was considered but ruled out by negative reverse transcriptase polymerase chain reaction for the Ewing sarcoma-related gene fusion product and Wilms’ tumor-related gene [11]. Low-grade HCC with neuroendocrine features, potentially a variant of fibrolamellar HCC, was suggested. However, the spindle-shaped epithelial cells and the lack of eosinophilic cells did not fit with that.

**Fig. 3:**
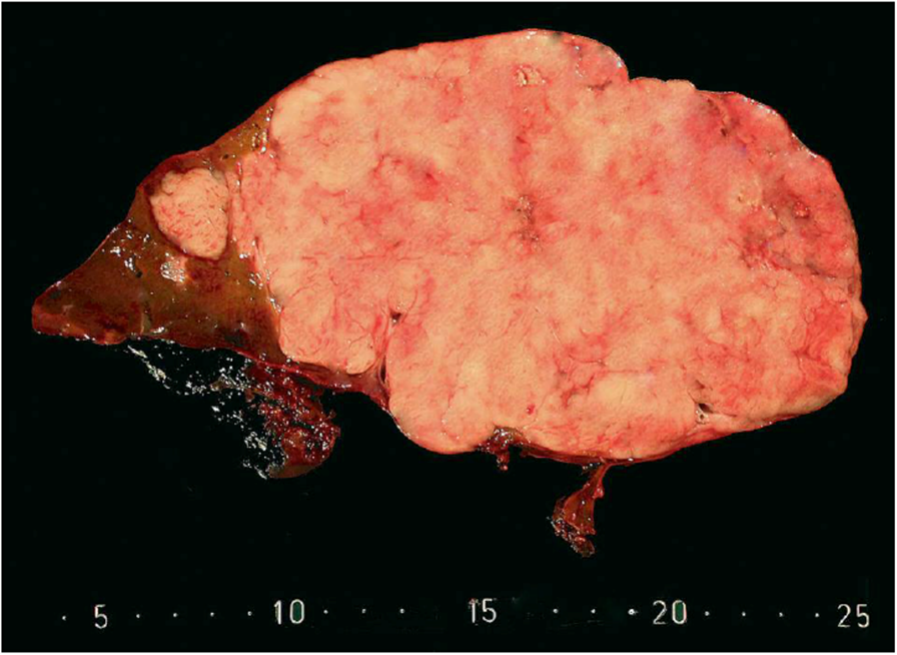
Macro image of the tumor showing a sharply demarcated multinodular mass of 19.5 cm with multiple small calcifications

**Fig. 4:**
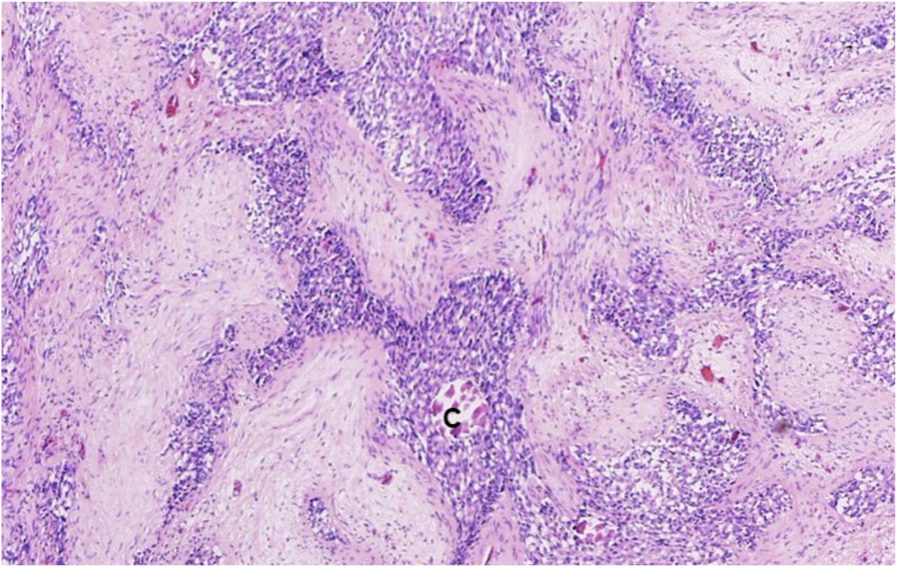
Microscopic image of the tumor showing psammomatous calcifications (*C*) within and near irregular nests of predominantly spindle-shaped epithelial cells

**Fig. 5:**
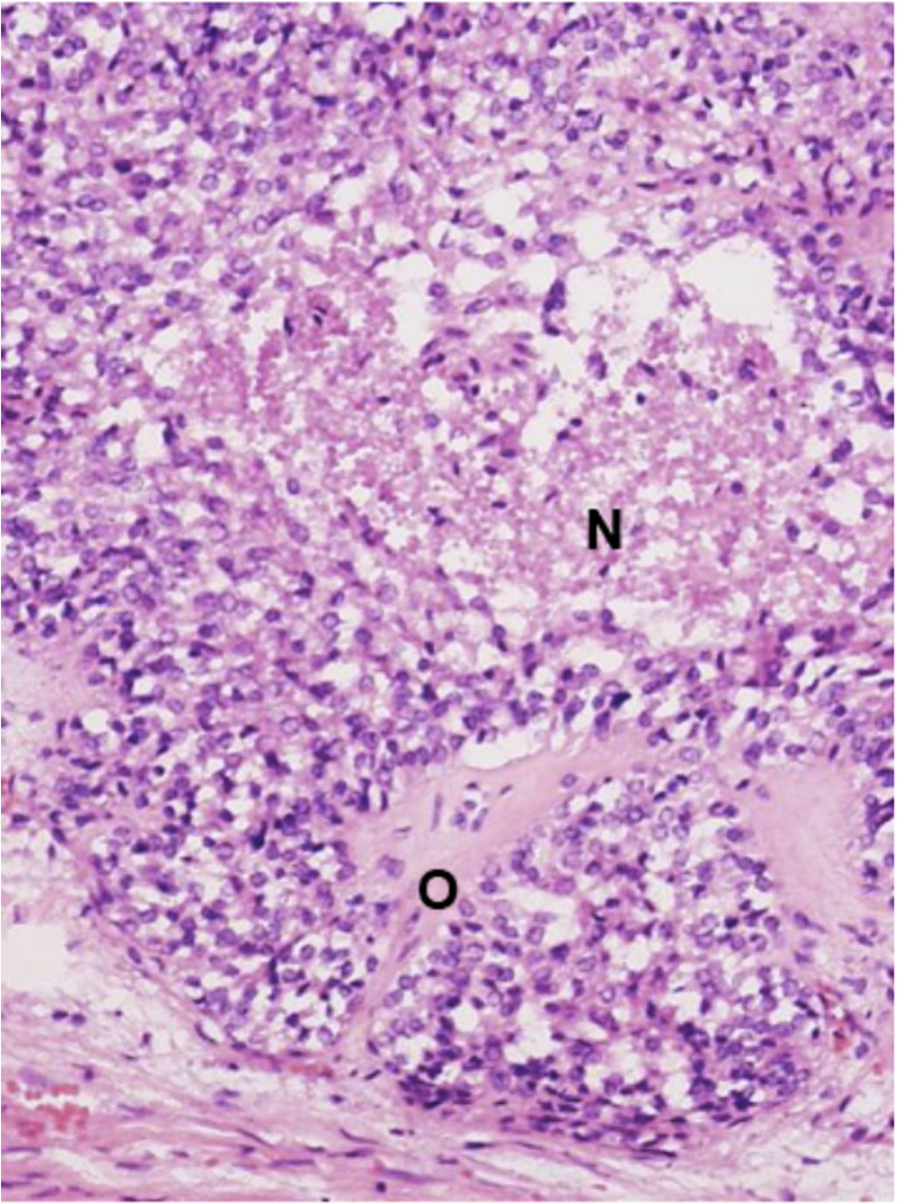
Microscopic image of the tumor showing epithelial cell nests with necrosis (*N*), cystic changes, and ossification (*O*) between the epithelial cells

On cut surface, a rather sharply demarcated multinodular mass of 19.5 cm with multiple small calcifications was found (Fig. 3). Histology confirmed an epithelial tumor. The size of the epithelial fields decreased and the degree of desmoplasia increased towards the center of the tumor. Psammomatous calcifications were found within and near irregular nests of predominantly spindle-shaped epithelial cells (Fig. 4). The nests showed necrosis and cystic changes. Ossification was seen between epithelial cells (Fig. 5). Nests of tumor cells were infiltrating the adjacent liver with entrapment of bile ducts and cords of hepatocytes. Few mitoses were seen. Comparison with the description of (C)NSET from the literature solved the diagnostic issue [1, 3]. Cushing syndrome in our patient was classified as ACTH-dependent and caused by an ectopic ACTH-secreting tumor.

## Discussion

We report the case of an adolescent patient with Cushing syndrome due to an ACTH-producing CNSET of the liver. While incidental cases such as the aforementioned hormone-producing hepatoblastoma (suggesting neuroendocrine differentiation) [9], and combined HCC and carcinoid [10] have been reported, overall, liver tumors producing hormones are rare. The differential diagnosis includes carcinoids, combined or coexisting HCC and neuroendocrine carcinoma, metastatic neuroendocrine tumor, metastatic primitive gonadal stromal tumor [5], and cystadenomas/cystadenocarcinomas with ovarian-like stroma [12]. These latter tumors express alpha-inhibin and are reactive to estrogen and progesterone receptors suggesting a common early fetal origin of the cystadeno(carcino)ma and ovarian-like stroma [12]. A special property of the liver tumor in our patient is that it produced a polypeptide hormone (ACTH). ACTH-producing tumors are more common in the thymus, thyroid, bronchus, lung, adrenals, and pancreas than in the liver [13, 14]. Localizations often suggest a neural crest origin [14]. These tumors may also produce insulin and catecholamines. Most likely, the CNSET in our patient belonged to this group.

Cushing syndrome has an annual incidence of two to five cases per million, of which children make up 10 % [15]. Ectopic ACTH-producing tumors account for 1 % of Cushing syndrome causes in adolescents [15]. Given the low incidence of primary hepatic ACTH-producing tumors, liver locations of ACTH-producing tumors are more likely to represent metastases from neuroendocrine tumors originating in other abdominal organs than primary tumors, rendering primary liver tumors causing Cushing syndrome as reported here extremely rare.

Surgical resection is the treatment of choice [13]. Cushing syndrome dissolves after complete resection of the causative tumor [13]. Liver transplant has been incidentally reported in the treatment of CNSET; two patients died of postoperative complications and consecutive lung metastases, respectively; another was without evidence of disease at 2-year follow-up [4–6]. The role of chemotherapy is unclear [1]. Histologic and immunological features of CNSET suggest a potential role for treatment directed at neuroendocrine tumors. A recent study shows altered mesenchymal–epithelial transition with deletions in the beta-catenin gene, suggesting another potential targeting route [7].

In general, in ectopic ACTH syndrome, histology of the causative tumor, dissemination of disease and control of hypercortisolemia influence morbidity and mortality [13]. In a series of 43 patients with ectopic ACTH-induced Cushing syndrome, median survival was 32 months with overall mortality rate of 63 % [14]. Progression of the causative malignancy and systemic infection were the leading causes of death [14].

CNSET has been suggested to have low malignant potential [1, 5, 7]. In that perspective, the level of aggression in treatment warranted is unclear. However, local recurrence after resection and re-recurrence after treatment of recurrence with radiofrequency ablation have been described [1–5]. Recently, extrahepatic lymph node and lung metastases were reported [4, 6]. However, the patient we present here is alive and well 13 years after complete resection. With few reported cases, the biological behavior of CNSET is difficult to predict.

Larger series through international collaboration such as Children’s Hepatic tumors International Collaboration (CHIC) of the Société Internationale d’Oncologie Pédiatrique – Epithelial Liver Tumor Study Group (SIOPEL), are needed before conclusions about epidemiology, treatment, and prognosis can be drawn.

## Conclusions

Despite its rarity, CNSET should be in the differential diagnosis of liver tumors, especially if associated with Cushing syndrome, as significant morbidity and mortality may be associated.

## Abbreviations

(C)NSET, (calcifying) nested stromal cell tumor; ACTH, adrenocorticotropic hormone; CHIC, Children’s Hepatic tumor International Collaboration; CNSET, calcifying nested stromal–epithelial tumor; HCC, hepatocellular carcinoma; MRI, magnetic resonance imaging; SIOPEL, Société Internationale d’Oncologie Pédiatrique – Epithelial Liver Tumor Study Group; SMA, smooth muscle actin
